# Etamycin as a Novel *Mycobacterium abscessus* Inhibitor

**DOI:** 10.3390/ijms21186908

**Published:** 2020-09-21

**Authors:** Bui Thi Bich Hanh, Tae Ho Kim, June-Woo Park, Da-Gyum Lee, Jae-Sung Kim, Young Eun Du, Chul-Su Yang, Dong-Chan Oh, Jichan Jang

**Affiliations:** 1 Molecular Mechanisms of Antibiotics, Division of Life Science, Research Institute of Life Science, Division of Applied Life Science (BK21plus Program), Gyeongsang National University, Jinju 52828, Korea; hanhm0515006@gstudent.ctu.edu.vn (B.T.B.H.); taeho12349@gmail.com (T.H.K.); sh050301@naver.com (D.-G.L.); 2 Department of Environmental Toxicology and Chemistry, Korea Institute of Toxicology, Jinju 52843, Korea; jwpark@kitox.re.kr; 3 Human and Environmental Toxicology Program, Korea University of Science and Technology (UST), Daejeon 34113, Korea; 4 Institute of Natural Science & Technology, Hanyang University, Ansan 15588, Korea; sung901017@naver.com; 5 Natural Products Research Institute, College of Pharmacy, Seoul National University, Seoul 08826, Korea; dye0302@snu.ac.kr (Y.E.D.); dongchanoh@snu.ac.kr (D.-C.O.); 6 Department of Molecular & Life Science, Hanyang University, Ansan 15588, Korea; chulsuyang@hanyang.ac.kr

**Keywords:** *Mycobacterium abscessus*, drug resistance, novel drug discovery

## Abstract

The increase in drug-resistant *Mycobacterium abscessus*, which has become resistant to existing standard-of-care agents, is a major concern, and new antibacterial agents are strongly needed. In this study, we introduced etamycin that showed an excellent activity against *M. abscessus*. We found that etamycin significantly inhibited the growth of *M. abscessus* wild-type strain, three subspecies, and clinical isolates in vitro and inhibited the growth of *M. abscessus* that resides in macrophages without cytotoxicity. Furthermore, the in vivo efficacy of etamycin in the zebrafish (*Danio rerio*) infection model was greater than that of clarithromycin, which is recommended as the core agent for treating *M. abscessus* infections. Thus, we concluded that etamycin is a potential anti-*M. abscessus* candidate for further development as a clinical drug candidate.

## 1. Introduction

Nontuberculous mycobacteria (NTM) is comprised of approximately 200 species, 95% of which are environmental bacteria that are not pathogenic to humans and animals. They are found widely in the human environment such as surface and tap water, soil, animals, milk, and food products [1]. However, some pathogenic NTM such as *Mycobacterium avium* complex (MAC) and *Mycobacterium abscessus* are of global importance in public health. Furthermore, human risk factors such as aging, pulmonary disease including cystic fibrosis (CF), and acquired immunodeficiency syndrome (AIDS), contribute to an increase in MAC and *M. abscessus* infection in many countries [2,3].

Of particular concern, *M. abscessus* infections are the most refractory. The clinical spectrum of *M. abscessus* has been categorized as pulmonary or extrapulmonary disease. Among them, chronic pulmonary infection with *M. abscessus* is seen in vulnerable hosts with CF, bronchiectasis, and chronic obstructive pulmonary disease (COPD), this infection is often incurable and associated with rapid lung function decline [4]. Furthermore, it requires a long-term antibiotic therapy, which often results in mortality [5]. In spite of its low virulence to humans compared to *Mycobacterium tuberculosis*, which causes human tuberculosis (TB), the *M. abscessus* lung infection is the most difficult to treat due to high levels of intrinsic drug resistance to current antibiotics, including the majority of β-lactams, tetracyclines, aminoglycosides, and macrolides. Moreover, anti-TB drugs, including rifampicin and isoniazid, are ineffective against *M. abscessus* [6]. The 2007 American Thoracic Society NTM guidelines noted that there is currently no drug regimen with proven efficacy against the pulmonary *M. abscessus* infection, but it suggested a macrolide (clarithromycin or azithromycin) combined with amikacin, and cefoxitin or imipenem as non-curative therapy [7,8]. Recent guidelines from the Center for Disease Control and Prevention recommended a regimen that also suggested the combination therapy with clarithromycin, amikacin, and cefoxitin as current antimicrobial drugs of choice for the treatment of *M. abscessus* infection [9]. However, these regimens are still under contention because they are ineffective and have considerable toxic side effects during the long course of treatment [7]. Thus, *M. abscessus* is an antibiotic nightmare indeed [10]. Therefore, there is an urgent need to discover new, effective drugs that are less toxic and more effective against *M. abscessus* lung disease.

Here, we report that a new anti-*M. abscessus* agent etamycin exhibits potent activity against *M. abscessus* in vitro, in infected murine macrophages, and zebrafish embryos.

## 2. Results

### 2.1. Chemical Structure of Etamycin

**Figure 1:**
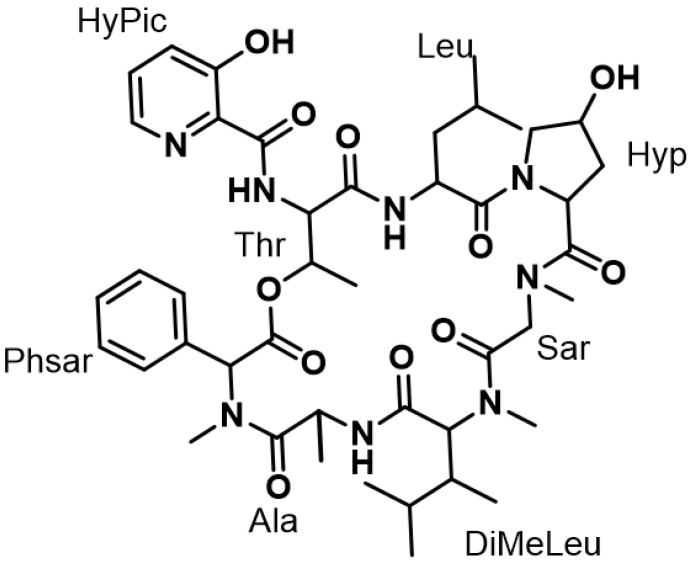
The planar structure of PHAR110904 (etamycin).

From the previous in-house screen, we have narrowed down one effective compound for *M. abscessus* with a cut-off of 80% inhibition at 20 μM (unpublished data). Among the screening hits, PHAR110904 (PHARMEKS LTD., code number) showed good in vitro activity. To validate the chemical structure of PHAR110904 that was provided from PHARMEKS LTD, we re-analyzed the chemical structure of PHAR110904 using NMR (nuclear magnetic resonance) spectroscopy. The molecular weight was confirmed as C_44_H_62_N_8_O_11_ based on the low resolution electrospray ionization mass spectrometric (LR-ESI-MS) data ([M + H]^+^ at *m*/*z* 879 and [M − H]^−^ at *m*/*z* 877) along with ^1^H and ^13^C NMR spectra. A comprehensive analysis of 1D and 2D NMR spectroscopic data, and eight main subunits of etamycin were established as phenylsarcosine (Phsar), alanine (Ala), dimethylleucine (DiMeLeu), sarcosine (Sar), hydroxyproline (Hyp), leucine (Leu), Threonine (Thr), and hydroxypicolinic acid (HyPic), identically to the planar structure of etamycin (Figure 1).

### 2.2. Etamycin Exhibits Potent Activity against M. abscesuss Subspecies, Clinical Isolates, and Drug-Resistant Strains

**Figure 2:**
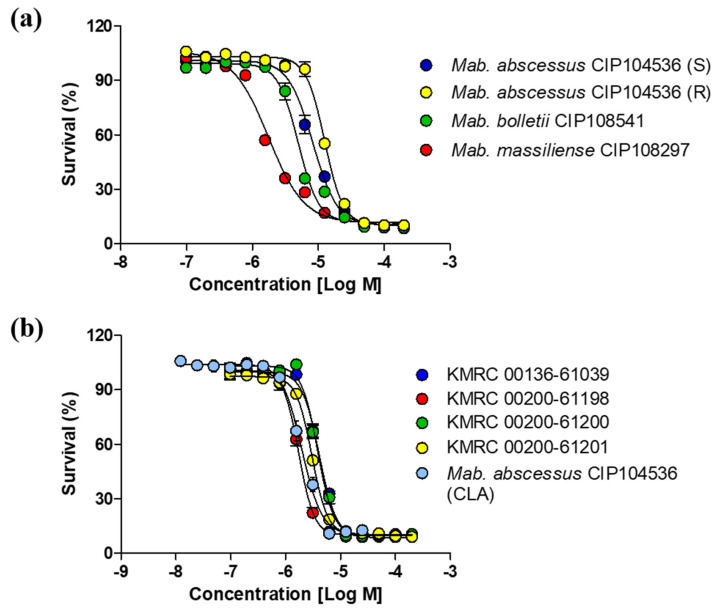
In vitro activity of etamycin for *M. abscessus* subspecies and clinical isolates. (**a**) Activity of etamycin against *M. abscessus* subsp. *abscessus* CIP 104536^T^ (S) and (R) morphotypes, *M. abscessus* subsp. *massiliense* CIP108297^T^, and *M. abscessus* subsp. *bolletii* CIP108541^T^ was evaluated in 7H9 medium by the resazurin microtiter assay (REMA). (**b**) Activity of etamycin against *M. abscessus* clinical isolates. Clarithromycin was used as a positive control for *M. abscessus* subsp. *abscessus* CIP 104536^T^ S morphotype. Dose-response curves were plotted using the GraphPad Prism software (version 6.05). Data are expressed as the mean ± standard deviation (SD) of triplicates for each concentration. CLA: Clarithromycin; KMRC: Korean Mycobacterial Resource Center.

To measure the activity of etamycin, we evaluated the minimum concentrations that caused 50% inhibition of growth of *M. abscessus* (MIC_50_) using etamycin for three different *M. abscessus* subspecies that comprise *M. abscessus* subsp. *abscessus* CIP 104536^T^ smooth (S) and rough (R) morphotypes, *M. abscessus* subsp. *massiliense* CIP108297^T^, and *M. abscessus* subsp. *bolletii* CIP108541^T^. Clarithromycin was selected as the positive control. After incubating *M. abscesuss* strains for 5 days with the compounds, a decrease in fluorescence was observed, indicating a dose-dependent killing effect. As shown in Figure 2a, all the subspecies tested were susceptible to etamycin. The range of MIC_50_ values of etamycin for strains was 1.8–8.2 μM (MIC_90_ value 4.3–28.3 μM). Moreover, *M. abscessus* subsp. *massiliense* CIP108297^T^ showed the lowest MIC_50_ value at 1.8 μM (MIC_90_ value 4.3 μM), and *M. abscessus* subsp. *abscessus* CIP 104536^T^ showed the highest MIC_50_ value at 8.2 μM (MIC_90_ value 28.3 μM); *M. abscessus* subsp. *bolletii* CIP108541^T^ was 5.0 μM (MIC_90_ value 16.0 μM). We also evaluated the etamycin activity against *M. abscesuss* clinical isolates that have a smooth (S) morphotype. The grouping of clinical isolates were previously confirmed by 16S rRNA gene sequencing, *rpoB*, and *hsp65* [11]. As shown in Figure 2b, a significant growth inhibition was observed when *M. abscesuss* clinical isolates were treated with various concentrations of etamycin. MIC_50_ values ranged from 1.7–4.1 μM (MIC_90_ value 4.3–10.3 μM). Clarithromycin was used as a positive control and it also inhibited the growth of *M. abscessus* subsp. *abscessus* CIP 104536^T^ with an MIC_50_ similar to etamycin for all the *M. abscessus* strains tested. Therefore, etamycin can be considered an effective drug candidate for drug-resistant strains.

### 2.3. Etamycin Inhibits the Growth of Intracellular M. abscessus without Significant Cytotoxicity

**Figure 3:**
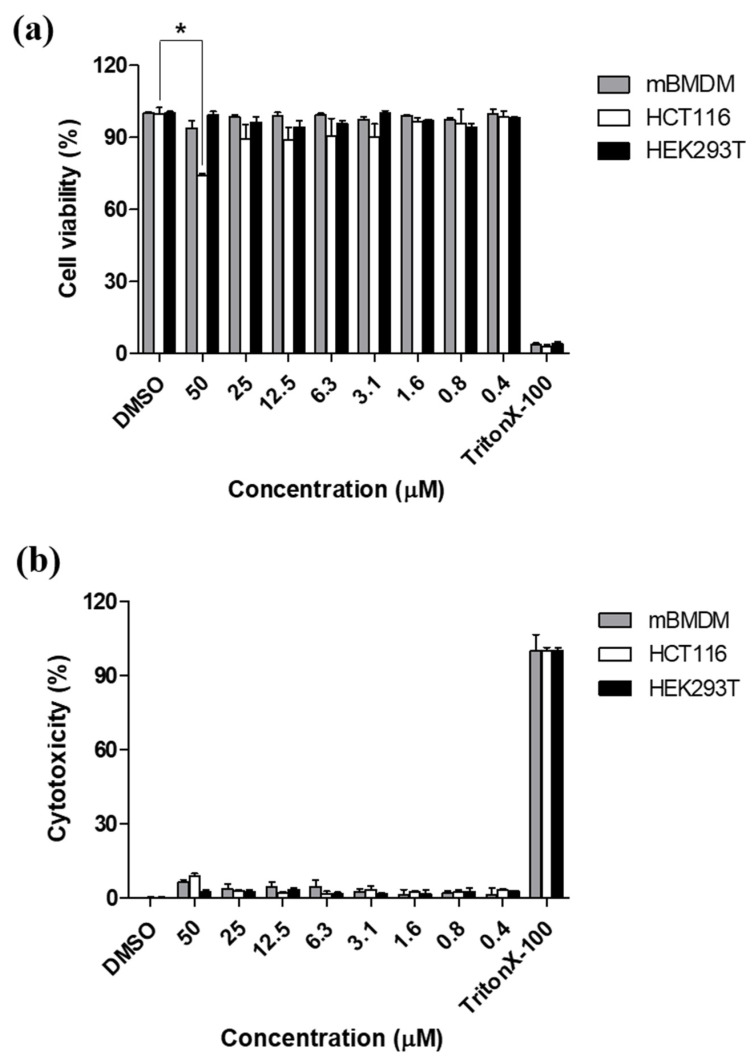
Etamycin effect on cell viability and cytotoxicity. Effect of etamycin on viability (**a**) and cytotoxicity (**b**) in murine bone marrow-derived macrophages (mBMDM), HCT116, and HEK293 cells was evaluated on day 3 after treatment with different concentrations of etamycin. Data were expressed as the mean ± SD of triplicates for each tested concentration. The relative difference (* p < 0.05) was compared with the DMSO control.

To determine whether etamycin influences the cell number, the cell viability at different concentrations of etamcyin was assessed 3 days after the treatment using the Cellrix^®^ Viability assay kit. The results of the effect of etamycin on the viability of murine bone marrow-derived macrophages (mBMDMs), human colon cancer cells (HCT116), and human embryonic kidney cells (HEK293) are presented in Figure 3a. No difference could be seen at any concentration for mBMDM and HEK293, whereas for HCT116 cells, the 50 μM (*p* < 0.05) groups had reduced cell viability compared with the DMSO control. Furthermore, to determine whether etamycin can enhance cell death in various cell types, we also examined the cytotoxicity of etamycin in mBMDM, HCT116, and HEK293 cells using the lactate dehydrogenase (LDH) assay after 3 days of treatment. As shown in Figure 3b, no significant cytotoxicity was observed in any of the cells tested. However, 1% Triton-X control showed approximately 100% cytotoxicity. These results suggest that etamycin does not affect the cell number and has no toxic effect on different cell types except for the highest concentration on the HCT116 cell line.

**Figure 4:**
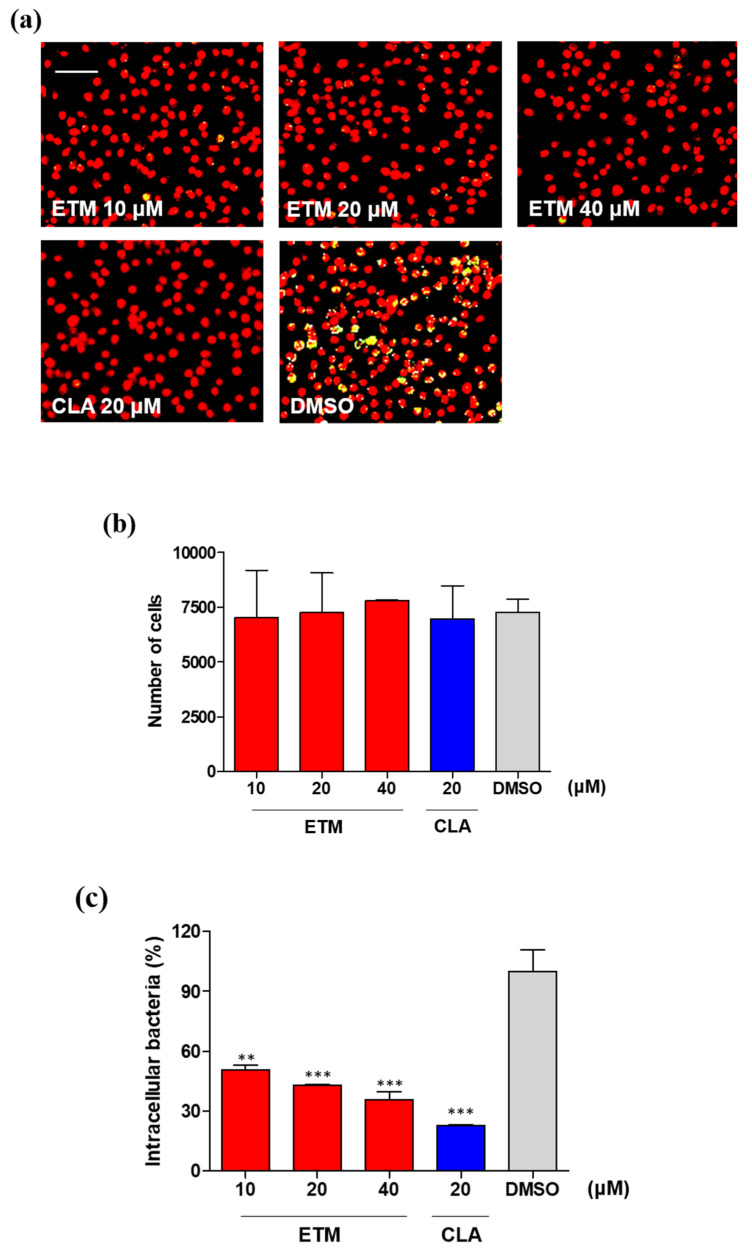
Etamycin intracellular activity in mWasabi protein-expressing *M. abscessus*-infected mBMDMs. (**a**) Images of mWasabi protein-expressing *M. abscessus* subsp. *abscessus* CIP104536^T^ (S) infected mBMDMs cells on day 3 after treatment with different concentrations of etamycin (10, 20, and 40 μM). DMSO and clarithromycin at 20 μM were used as negative and positive controls, respectively. After 3 days, cells were stained with syto60 (red), and the cells were analyzed using the automated cell imaging system. The yellow colors indicated mWasabi protein-expressing *M. abscessus* that were phagocytized by red-stained mBMDMs. (**b**) The cell number of *M. abscessus*-infected mBMDMs was enumerated after treatment with each compound. (**c**) Pixel intensities of mWasabi-expressing *M. abscessus* in mBMDMs were quantified after treatment of cells with different concentrations of etamycin and were compared with those of negative (DMSO) and positive (clarithromycin; 20 µM) controls. The relative difference (** *p* < 0.01; *** *p* < 0.001) was compared with the DMSO control. Data are expressed as the mean ± SD of duplicates for each concentration. CLA: Clarithromycin and ETM: Etamycin. Scale bar: 55.2 µm.

In order to further validate the activity of etamycin, we tested the potency of etamycin using a macrophage-based phenotypic assay with an automated cell imaging system to monitor the intracellular growth of mWasabi protein-expressing *M. abscessus* subsp. *abscessus* CIP104536^T^ (S) in mBMDMs. mBMDM cells were seeded at 6 × 10^5^ per well and infected with mWasabi protein-expressing *M. abscessus* that were mixed at a multiplicity of infection (MOI) of 10:1. Around 100% of the cells were infected by mWasabi protein-expressing *M. abscessus* subspecies *abscessus* CIP 104536T (S) (Figure S1). Clarithromycin and DMSO were used as positive and negative controls, respectively. After adding each compound to the mWasabi protein-expressing *M. abscessus*-infected mBMDMs cells in a dose-dependent manner, the culture plates were incubated for 3 days at 37 °C, after which images were acquired and analyzed. The CellReporterXpress^®^ Image Acquisition and Analysis Software was used to quantify several different parameters such as the number of host macrophages, percentage of infected cells, and total fluorescent intensity. As shown in Figure 4a, etamycin showed significant activity against *M. abscessus* replication in macrophages. More precisely, host cells treated with 10, 20, and 40 μM of etamycin harbored significantly fewer bacteria than those treated with the DMSO control. Furthermore, no reduction in cell number was observed after etamycin treatment at any tested concentration (Figure 4b). Finally, the percentage of pixel intensity of intracellular mWasabi protein-expressing *M. abscessus* at different concentrations of etamycin was compared with that of the positive and negative controls. As shown in Figure 4c, etamycin-treated mBMDMs showed a significantly reduced mWasabi pixel intensity in a concentration-dependent manner compared to the DMSO control-treated cells. Clarithromycin also demonstrated good activity against *M. abscessus* at 20 μM. However, many intracellular mWasabi protein-expressing *M. abscessus* were quantified in DMSO control-treated cells. This demonstrates that etamycin can enter the host cell membrane and inhibit bacterial growth inside the cell.

### 2.4. Etamycin Shows Antimicrobial Activity against Rough Morphotype of M. abscessus in Zebrafish

**Figure 5:**
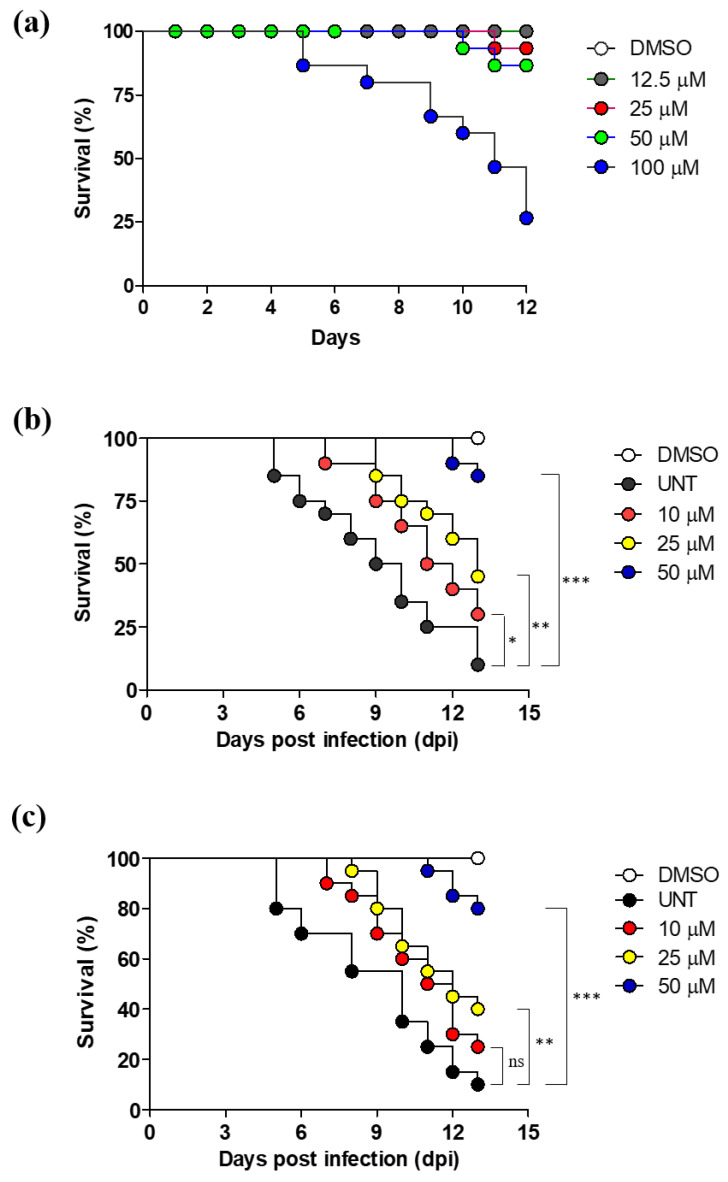

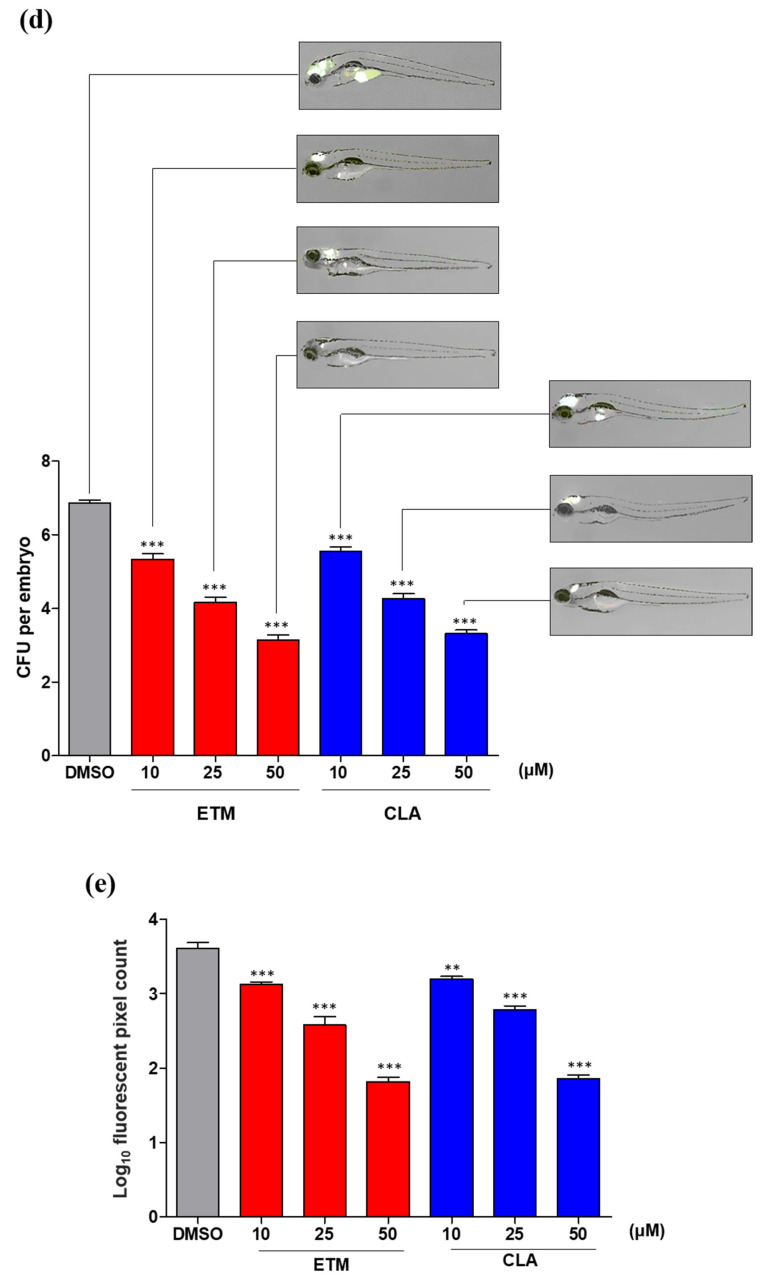
In vivo efficacy of etamycin in the zebrafish (ZF) model of infection. (**a**) Various concentrations of the etamycin (12.5, 25, 50, and 100 μM) were treated to the zebrafish without *M. abscessus* infection and the maximum tolerated dose of etamycin was measured. (**b**) The survival curve was plotted from *M. abscessus* CIP 104536^T^ (R)-infected embryos treated at 10, 25, and 50 μM of etamycin. (**c**) The survival curve was generated from *M. abscessus* CIP 104536^T^ (R)-infected embryos treated at 10, 25, and 50 μM of clarithromycin (*n* = 20, representative of three independent experiments). Survival curves were compared with the log-rank (Mantel-Cox) test (* *p* < 0.05, ** p < 0.01; *** p < 0.001; ns: Not significant). (**d**) Evaluation of the in vivo etamycin activity on mWasabi protein-expressing *M. abscessus* CIP 104536^T^ (R) infection. 400 colony-forming units (CFU) was infected via the caudal vein infection and visualized in untreated or etamycin-treated embryos under the fluorescent microscope. Bacterial burden of untreated etamycin and clarithromycin treated embryos was enumerated and expressed as the mean log10 CFU per embryo (*n* = 5 of each condition) from three independent experiments. (**e**) Comparison of bacterial quantification by the fluorescent pixel count (FPC) in etamycin (ETM) and clarithromycin treated zebrafish at 5 dpi. Results are expressed as the mean log_10_ FPC (*n* = 5 of each condition). The relative difference (*** p < 0.001) was compared with the DMSO control. CLA: Clarithromycin; ETM: Etamycin; and UNT: Untreated control.

In order to determine the highest dose at which etamycin can be administered without lethality, we evaluated the maximum tolerated dose (MTD) of etamycin to zebrafish (ZF). Fifteen ZF were used in each treatment group. A broad range of etamycin (ranging from 12.5 to 100 μM) was added to zebrafish-containing fish water, without bacterial infection. As shown in Figure 5a, 76% of fish died after 12 days of exposure to 100 μM etamycin, while above 87% of zebrafish survived at lower concentrations (12.5, 25, and 50 μM). Thus, less than 50 µM etamycin, which does not show significant lethality, were used for in vivo efficacy tests.

To determine the therapeutic potential of etamycin, the drug in vivo efficacy was evaluated in zebrafish after infection with mWasabi protein-expressing *M. abscessus,* as described previously [11]. As *M. abscessus* CIP 104536^T^ (R) is hypervirulent in animal models, we evaluated the activity of etamycin against *M. abscessus* CIP 104536^T^ (R) at concentrations of 10, 25, and 50 µM [12]. As the first approach, we investigated whether increasing the etamycin dose would consequentially expand the lifespan of *M. abscessus* CIP 104536^T^(R)-infected ZF. For this, the percentage survival of ZF was monitored using the Kaplan–Meier method for 13 dpi. The etamycin contained fish water that was replaced daily. As shown in Figure 5b, approximately 90% of fish in the untreated group died at 13 dpi. However, the etamycin treated group resulted in significantly increased ZF lifespan in a dose-dependent manner when *M. abscessus* CIP 104536^T^(R)-infected ZF was exposed to 10, 25, and 50 μM for 13 days. Survival of the ZF was 30% and 45% at 10 and 25 μM of etamycin, respectively, but it increased exponentially to 85% at 50 μM of etamycin, greater than that of clarithromycin observed at 50 μM (Figure 5c).

Second, the mWasabi protein-expressing *M. abscessus* CIP 104536^T^(R) dissemination in ZF was observed under a fluorescence microscope. After the bacterial infection, etamycin was administered at three different concentrations (10, 25, and 50 μM) up to 5 dpi (days post-infection). As shown in Figure 5d, mWasabi protein-expressing *M. abscessus* CIP 104536^T^(R) showed a substantial increase, mainly within the developing ZF brain and yolk sac extension areas, in the DMSO control. However, surprisingly, the etamycin treatment in ZF during mWasabi protein-expressing *M. abscessus* CIP 104536^T^ (R) infection significantly reduced the degree of green fluorescence. A significant mWasabi protein reduction was observed within the ZF brain and yolk sac extension when the infected ZF embryos were treated with 10 μM etamycin, and only a small mWasabi protein signal could be detected in the ZF brain when ZF were treated with 50 μM etamycin. Treatment with the positive control, clarithromycin, also showed a significant reduction at 5 dpi in ZF (Figure 5d).

Next, the growth of *M. abscessus* CIP 104536^T^ (R) in the etamycin-treated ZF was enumerated through quantification of colony-forming units (CFU) per embryo. After 5 days of treatment, a statistically significant reduction was observed in the number of CFU/embryo at 10, 25, and 50 μM of etamycin, demonstrating that etamycin inhibits *M. abscessus* CIP 104536^T^ (R) replication in ZF. The CFU/embryo of etamycin observed at 50 μM was comparable to that of clarithromycin (50 μM concentration) (Figure 5d).

In parallel, images of the mWasabi-expressing *M. abscessus*-infected ZF were captured for determining the bacterial dissemination in ZF after treatment with etamycin; the bacterial dissemination was analyzed by quantification of fluorescent pixel count (FPC), which reflects the bacterial burden inside the ZF, using ImageJ software. As shown in Figure 5e, a significant decrease in the bacterial burden was observed in etamycin- and clarithromycin-treated cells, suggesting that these two compounds showed a similar in vivo efficacy as shown in CFU enumeration (Figure 5d). Taken together, these results suggest that etamycin exhibits a therapeutic effect against *M. abscessus* in vivo.

## 3. Discussion

*M. abscessus* is a notorious drug-resistant mycobacterial species, and there is a lack of new active molecules. The current clarithromycin-based treatment regimens are only moderately effective, and clarithromycin resistance in some isolates consequently causes treatment failure [13,14]. Thus, finding new chemical entities that have potent activity against *M. abscessus* is of vital importance. Consequently, several different drug discovery approaches have been recently conducted, such as whole-cell screening, new combination studies that cause synergistic effects with already existing drugs or drug re-positioning studies with old drugs [15]. However, there is still a poor promising new chemical lead that is waiting for clinical phase III and market release [16]. Currently, only three clinical phase II studies have been completed to determine safety, tolerance, and efficacy. First, tigecycline was also completed in a Phase II clinical trial in 2010. Second, an inhaled formulation of nitric oxide (NO) has been performed in phase II of clinical development for the treatment of *M. abscessus* and other non-tuberculous mycobacteria in 2019. Lastly, the novel formulation of liposomal amikacin for inhalation (LAI) that reduced toxicity and improved effectiveness for *M. abscessus* also completed clinical phase II in 2019. However, as yet, no study results have been posted on ClinicalTrials.gov (https://clinicaltrials.gov/) for all these clinical trials [16,17]. To make matters worse, there are not many active new drug candidates in clinical phase I studies, even at the discovery level. This may be due to the extremely low hit rate in a chemical drug screen targeting *M. abscessus* [18,19,20,21,22]. Therefore, identification of any active agent for *M. abscessus* is urgently required, although the minimum inhibitory concentration (MIC) value is in the micromole range.

Etamycin has been shown to have potent activity against several different pathogens, such as methicillin-resistant *Staphylococcus aureus* (MRSA), *Streptococcus pyogenes,* and *Streptococcus agalactiae*. Etamycin was also active against the Gram-negative respiratory tract pathogens *Moraxella catarrhalis* and *Haemophilus influenzae* [23]. Recently, Hosoda et al. demonstrated the anti-mycobacterial activity of etamycin against *Mycobacterium avium* and *Mycobacterium intracellulare* at a very low MIC concentration range (0.024–1.56 µg/mL). Furthermore, it showed enhanced anti-mycobacterial activity through combination with griseoviridin in a silkworm infection assay against *Mycobacterium smegmatis* [24]. The mechanism of action of etamycin hypothesized that it may inhibit MRSA via inhibition of the protein synthesis [25].

In this study, we evaluated the activity of etamycin in three different ways and showed a similar anti-*M. abscessus* activity in all three models. First, we tested the in vitro susceptibility of *M. abscessus* to etamycin. As shown in Figure 2a,b, the survival of all three *M. abscessus* subspecies was greatly decreased in the presence of etamycin and in vitro activity comparable to that of clarithromycin. Furthermore, we evaluated the antibacterial activity of etamycin against clinical strains of *M. abscessus* and found that etamycin was equally effective against *M. abscessus* clinical isolates from different sources, selected from the Korean Mycobacterial Resource Center (KMRC).

Second, the intracellular antimicrobial activity of etamycin against *M. abscessus,* which is replicated inside murine bone marrow-derived macrophages (mBMDMs) was assessed. During infection, *M. abscessus* is phagocytized by macrophages and replicates inside immune cells via overcoming host defenses [26]. Therefore, the identification of compounds that can kill intracellular *M. abscessus* through penetration of the cell membrane is crucial. For this reason, we developed a method to observe live fluorescent *M. abscessus* dissemination and inhibition inside mBMDMs using an automated cell imaging system. For evaluation, mBMDMs were stained with syto60 after infection with mWasabi protein-expressing *M. abscessus*, and bacterial growth was observed and statistically quantified based on the emission of mWasabi from *M. abscessus* and signal of syto60 from mBMDMs. Using this system, we evaluated the intracellular activity and cytotoxicity of etamycin in mBMDMs at the same time. As shown in Figure 4a, etamycin showed good inhibitory effects at 10 μM, without cytotoxicity. Moreover, etamycin did not show a significant reduction of cell viability and cytotoxicity to various cell lines up to 50 μM (Figure 3). Thus, we demonstrate that etamycin can be delivered into host cells without cytotoxicity and is an effective agent for inhibiting the growth of intracellular *M. abscessus*.

Lastly, this therapeutic activity was also confirmed in vivo using zebrafish (ZF). ZF is a popular animal model for in vivo antitubercular drug discovery [11,15,22,26]. In particular, this model is a very good tractable model for *M. abscessus* and *M. marinum* that can survive and replicate at cold environments (~30 °C). Furthermore, a larval zebrafish-mycobacterial infection platform for drug candidates has been validated as an anti-bacterial activity in ZF, showing preliminary in vivo efficacy before proceeding to a mouse model. In a similar manner, we also evaluated the in vivo efficacy of etamycin by injecting *M. abscessus* subsp. *abscessus* CIP 104536^T^ (R) into ZF using the survival curve and CFU enumeration. In this in vivo efficacy test, ~400 CFU *M. abscessus* resulted in 90% of ZF death infected at 13 dpi, while microinjection of equal numbers of *M. abscessus* with the etamycin treatment resulted in a *significantly enhanced lifespan. For example*, *85% of M. abscessus* infected ZF that were treated with 50 μM etamycin survived until 13 dpi. This result showed greater in vivo efficacy compared to clarithromycin, which is recommended as the core agent for treating *M. abscessus* infections at the same concentration (~80% survival at 13 dpi).

In this study, we report the anti-*M. abscessus* activity of etamycin. We concluded that etamycin was highly effective against wild-type and clinical isolates of *M. abscessus* in vitro, and it is also effective for replicating *M. abscessus* in mBMDMs. Furthermore, etamycin also showed its anti-*M. abscessus* activity for *M. abscessus* CIP 104536^T^ (R), which tends to be more virulent in ZF in vivo efficacy tests. Thus, etamycin is a promising new inhibitor that can be further developed for the treatment of *M. abscessus* infections.

## 4. Materials and Methods

### 4.1. Chemical Analysis Using Nuclear Magnetic Resonance Spectroscopy

All NMR spectra (^1^H, ^13^C, HSQC, HMBC, and COSY) were acquired using a Bruker Avance spectrometer (Bruker, Billerica, MA, USA) (Research Institute of Pharmaceutical Sciences in Seoul National University) at 800 and 200 MHz for ^1^H and ^13^C NMR, respectively. ^1^H and ^13^C chemical shifts were referenced with the DMSO-*d*_6_ solvent peaks at *δ*_H_ 2.50 and 39.5, respectively. A low resolution electrospray ionization mass spectrometric (LR-ESI-MS) analysis was performed with an Agilent Technologies 1200 series HPLC connected with an Agilent Technologies 6140 quadrupole mass spectrometer.

### 4.2. Bacterial Strains and Culture Conditions

*M. abscessus* subsp. *abscessus* CIP 104536^T^ S- and R-morphotypes were kindly provided by Dr. Laurent Kremer (CNRS, IRIM, Universite’ de Montpellier, Montpellier, France). *M. abscessus* subsp. *bolletii* CIP108541^T^ and *M. abscessus* subsp. *massiliense* CIP108297^T^ were obtained from the Collection de l’Institut Pasteur (CIP, Paris, France). Clinical isolates were purchased from the Korea Mycobacterium Resource Center (KMRC, Osong, Korea). *M. abscessus* strains were grown as described previously without modification [22]. Clarithromycin was purchased from Sigma-Aldrich (St. Louis, MO, USA), and etamycin (code number, PHAR110904) was purchased from PHARMEKS (Moscow, Russia). For the murine macrophage and zebrafish embryo infection, recombinant *M. abscessus* CIP 104536^T^ carrying a pMV262-mWasabi plasmid expressing mWasabi protein was prepared as previously described [11].

### 4.3. Resazurin Microtiter Assay (REMA)

For drug susceptibility testing, REMA was performed under aerobic conditions. The resazurin solution was prepared as a 0.025% (wt/vol) solution in sterile distilled water using resazurin sodium salt powder (Sigma, St. Louis, MO, USA), filter sterilized. Bacteria from exponential-phase cultures were harvested and adjusted to an OD590 of 0.05 (approximately 5 × 10^4^ CFU/mL) in wells of a 96-well microtiter plate. Two-fold serial dilutions of compounds were prepared from 200 μM to 97 nM etamycin and from 50 μM to 24 nM clarithromycin. Plates were then incubated at 37 °C. After 3 days of incubation, 40 μL of the resazurin solution was added to the wells. Fluorescence was measured using a SpectraMax^®^ M3 Multi-Mode Microplate Reader (Molecular Devices, Sunnyvale, CA, USA). Concentrations required to inhibit bacterial growth by 50% (IC_50_) were determined by fitting the curves with a sigmoidal dose-response using the GraphPad Prism software (version 6.05; San Diego, CA, USA).

### 4.4. Intracellular Bacterial Replication Assays

Cells were grown at 37 °C and 5% CO_2_ in Dulbecco’s Modified Eagle’s Medium (DMEM) high-glucose medium (LM001-05; Welgene, Gyeongsan-si, Gyeongsangbuk-do, Korea) supplemented with 10% heat-inactivated fetal bovine serum (S001-01; Welgene, Gyeongsan-si, Gyeongsangbuk-do, Korea). Murine monocytes were obtained by flushing femurs of six week-old female C57BL/six mice (Orient Bio, Seongnam-si, Gyeonggi-do, Korea) with DMEM-FBS. Monocytes were differentiated into mBMDM by exposure to a 40 ng/mL recombinant mouse macrophage colony-stimulating factor (M-CSF, JW-M003-0025; JW CreaGene, Seongnam-si, Gyeonggi-do, Korea) for 7 days. Ten thousand U/mL penicillin/streptomycin (15140-122; Gibco, Life Technologies Corporation, Grand Island, NY, USA) mixture was used for contamination. For the assessment of the activity of the etamycin against intracellular *M. abscessus*, mBMDMs (6 × 10^5^ cells/well) cells were infected with mWasabi protein-expressing *M. abscessus* subspecies *abscessus* CIP 104536^T^ (S) at a multiplicity of infection (MOI) of 10:1. After 3 h of infection, the cells were washed with PBS (phosphate-buffered saline) to remove the uninfected bacteria and incubated with DMEM (Dulbecco’s Modified Eagle’s medium) containing 250 μg/mL amikacin for 1 h to kill the remaining extracellular *M. abscessus*. The cells were washed with PBS and then dispensed into 96-well plates (Corning, New York, NY, USA). The broth medium, with a 2-fold serial dilution of etamycin, was subjected to a 96-well plate. For all experiments, the amount of DMSO was maintained at a 1% final concentration per well. After three days of incubation at 37 °C and 5% CO_2_, macrophages were stained for 1 h with 5 μM Syto 60 dye (Thermo Fisher Scientific, Waltham, MA, USA), and images were acquired using the ImageXpress Pico Automated Cell Imaging System (Molecular Devices, Sunnyvale, CA, USA). The bacterial load and macrophage number were quantified using the CellReporterXpress^®^ Image Acquisition and Analysis Software (Molecular Devices, Sunnyvale, CA, USA).

### 4.5. Cell Viability Assay and Lactate Dehydrogenase (LDH) Cytotoxicity Assay

The cell viability and cytotoxicity of etamycin were evaluated in mBMDM, HCT116, and HEK293 cells using the Cellrix^®^ Viability assay kit (Medifab, Seoul, Korea) and CytoTox 96^®^ Non-Radioactive Cytotoxicity Assay (Promega, Madison, WI, USA), respectively. HCT116 and HEK293 cells were grown at 37 °C and 5% CO_2_ in DMEM medium (Welgene) supplemented with a 5% heat-inactivated fetal bovine serum (FBS; Gibco, Life Technologies Corporation, Grand Island, NY, USA) for 2 days. The mBMDMs were placed into a 96-well plate (6.0 × 10^5^ cells/well) and incubated at 37 °C for 72 h. HCT116 and HEK293 cells were placed into a 96-well plate (1.0 × 10^4^ cells/well) and incubated at 37 °C for 72 h. Etamycin was added to the cells at various concentrations. For the cell viability assay, 10 μL of reagent (10% media volume) was added to each well after an additional 72 h of incubation, and the incubation was continued for 4 h. The resulting color was assayed at 450 nm. For the LDH cytotoxicity assay, 100 μL of reagent (10% media volume) was added to each well, and the incubation was continued for 30 min. The resulting color was assayed at 490 nm. Cells treated with 1% Triton-X-100 were used as a positive control, while 1% of the DMSO-treated cells were used as a negative control in both experiments. SpectraMax^®^ M3 Multi-Mode Microplate Reader (Molecular Devices, Sunnyvale, CA, USA) was used for each assay.

### 4.6. Ethics

All ZF experiments were approved by the Animal Research Ethics Committee of Gyeongsang National University (Project identification code: GNU-190325-E0014, Approval date: 25 Mar 2019).

### 4.7. Drug Efficacy Assessment in mWasabi Protein-Expressing M. abscessus-Infected Zebrafish

Zebrafish larvae at 30–48 h post-fertilization were dechorionated and anesthetized with 270 mg/L tricaine, and then infected with 3 nL containing approximately 400 CFU of mWasabi protein-expressing *M. abscessus* subspecies *abscessus* CIP 104536^T^ (R) via the caudal vein using a Nanoject III microinjector (Drummond Scientific, Broomall, PA, USA). The infected larvae were transferred into 96-well plates (two fishes/well) and incubated at 28.5 °C. Etamycin and clarithromycin at the final concentrations of 10, 25, and 50 µM were applied directly into the fish water (methylene blue 300 µL/L). The fish water and compound were renewed once daily. The infected untreated embryos served as a negative control. The in vivo assessment efficacy of drugs was determined by following the bacterial burden, mWasabi protein quantification, and the kinetics of embryo survival. For the bacterial load quantification, groups of three infected embryos (5 dpi) were collected and individually homogenized in PBS with 2% Triton-X 100 using a handheld homogenizer (D1000; Benchmark Scientific, Sayreville, NJ, USA). Serial 10-fold dilutions of the suspension were plated on the Middlebrook 7H10 agar medium supplemented with 10% oleic acid-albumin-dextrose-catalase (OADC; Difco, Detroit, MI, USA) containing 50 µg/mL kanamycin and BBL mycobacteria growth indicator tubes MGIT PANTA (polmyxin B, amphotericin B, nalidixic acid, trimethoprim, and azlocillin; Becton Dickinson, Franklin Lakes, NJ, USA) and then incubated for 5 days at 37 °C to enumerate the CFU. The mWasabi protein quantification was assessed by observing the mWasabi evolution using a SteREO Lumar V12 stereomicroscope with fluorescence optics (Zeiss, Jena, Germany). Dead embryos (no heartbeat) were recorded daily for 13 days to determine the survival curve. The survival curve was plotted by Prism using the Kaplan–Meier method and log-rank (Mantel–Cox) test to compare the difference between untreated, control, and treated embryos. The drug efficacy was evaluated via the measurement of fluorescent pixel count (FPC) using the “Analyse particles” function in ImageJ, as described previously [26].

## Supplementary Materials

It is available online at https://www.mdpi.com/1422-0067/21/18/6908/s1.

## References

[1] de Ruyck J., Dupont C., Lamy E., Le Moigne V., Biot C., Guérardel Y., Herrmann J.L., Blaise M., Grassin-Delyle S., Kremer L. (2020). Structure-Based Design and Synthesis of Piperidinol-Containing Molecules as New Mycobacterium abscessus Inhibitors. ChemistryOpen.

[2] Johansen M.D., Herrmann J.L., Kremer L. (2020). Non-tuberculous mycobacteria and the rise of Mycobacterium abscessus. Nat. Rev. Microbiol..

[3] Nishiuchi Y., Iwamoto T., Maruyama F. (2017). Infection sources of a common non-tuberculous mycobacterial pathogen, Mycobacterium avium complex. Front. Med..

[4] Maggioncalda E.C., Story-Roller E., Mylius J., Illei P., Basaraba R.J., Lamichhane G. (2020). A mouse model of pulmonary Mycobacteroides abscessus infection. Sci. Rep..

[5] Chen J., Zhao L., Mao Y., Ye M., Guo Q., Zhang Y., Xu L., Zhang Z., Li B., Chu H. (2019). Clinical efficacy and adverse effects of antibiotics used to treat mycobacterium abscessus pulmonary disease. Front. Microbiol..

[6] Luthra S., Rominski A., Sander P. (2018). The role of antibiotic-target-modifying and antibiotic-modifying enzymes in mycobacterium abscessusdrug resistance. Front. Microbiol..

[7] Berube B.J., Castro L., Russell D., Ovechkina Y., Parish T. (2018). Novel screen to assess bactericidal activity of compounds against non-replicating mycobacterium abscessus. Front. Microbiol..

[8] Daniel-Wayman S., Shallom S., Azeem N., Olivier K.N., Zelazny A.M., Prevots D.R. (2019). Amikacin exposure and susceptibility of macrolide-resistant Mycobacterium abscessus. ERJ Open Res..

[9] Lee M.-R., Sheng W.-H., Hung C.-C., Yu C.-J., Lee L.-N., Hsueh P.-R. (2015). Mycobacterium abscessus Complex Infections in Humans. Emerg. Infect. Dis..

[10] Nessar R., Cambau E., Reyrat J.M., Murray A., Gicquel B. (2012). Mycobacterium abscessus: A new antibiotic nightmare. J. Antimicrob. Chemother..

[11] Kim T.H., Bich Hanh B.T., Kim G., Lee D.G., Park J.W., Lee S.E., Kim J.S., Kim B.S., Ryoo S., Jo E.K. (2019). Thiostrepton: A Novel Therapeutic Drug Candidate for Mycobacterium abscessus Infection. Molecules.

[12] Catherinot E., Clarissou J., Etienne G., Ripoll F., Emile J.F., Daffé M., Perronne C., Soudais C., Gaillard J.L., Rottman M. (2007). Hypervirulence of a rough variant of the Mycobacterium abscessus type strain. Infect. Immun..

[13] Benwill J.L., Wallace R.J. (2014). Mycobacterium abscessus: Challenges in diagnosis and treatment. Curr. Opin. Infect. Dis..

[14] Koh W.-J. (2017). Nontuberculous Mycobacteria—Overview. Microbiol. Spectr..

[15] Dupont C., Viljoen A., Dubar F., Blaise M., Bernut A., Pawlik A., Bouchier C., Brosch R., Guérardel Y., Lelièvre J. (2016). A new piperidinol derivative targeting mycolic acid transport in Mycobacterium abscessus. Mol. Microbiol..

[16] Wu M.L., Aziz D.B., Dartois V., Dick T. (2018). NTM drug discovery: Status, gaps and the way forward. Drug Discov. Today.

[17] Degiacomi G., Sammartino J.C., Chiarelli L.R., Riabova O., Makarov V., Pasca M.R. (2019). Mycobacterium abscessus, an emerging and worrisome pathogen among cystic fibrosis patients. Int. J. Mol. Sci..

[18] Gupta R., Netherton M., Byrd T.F., Rohde K.H. (2017). Reporter-based assays for high-throughput drug screening against Mycobacterium abscessus. Front. Microbiol..

[19] Jeong J., Kim G., Moon C., Kim H.J., Kim T.H., Jang J. (2018). Pathogen Box screening for hit identification against Mycobacterium abscessus. PLoS ONE.

[20] Chopra S., Matsuyama K., Hutson C., Madrid P. (2011). Identification of antimicrobial activity among FDA-approved drugs for combating Mycobacterium abscessus and Mycobacterium chelonae. J. Antimicrob. Chemother..

[21] Malin J.J., Winter S., Van Gumpel E., Plum G., Rybniker J. (2019). Extremely low hit rate in a diverse chemical drug screen targeting mycobacterium abscessus. Antimicrob. Agents Chemother..

[22] Hanh B.T.B., Park J.W., Kim T.H., Kim J.S., Yang C.S., Jang K., Cui J., Oh D.C., Jang J. (2020). Rifamycin O, An Alternative Anti-Mycobacterium abscessus Agent. Molecules.

[23] Haste N.M., Perera V.R., Maloney K.N., Tran D.N., Jensen P., Fenical W., Nizet V., Hensler M.E. (2010). Activity of the streptogramin antibiotic etamycin against methicillin-resistant Staphylococcus aureus. J. Antibiot. (Tokyo).

[24] Hosoda K., Koyama N., Kanamoto A., Tomoda H. (2019). Discovery of Nosiheptide, Griseoviridin, and Etamycin as Potent Anti-Mycobacterial Agents against Mycobacterium avium Complex. Molecules.

[25] Schinke C., Martins T., Queiroz S.C.N., Melo I.S., Reyes F.G.R. (2017). Antibacterial Compounds from Marine Bacteria, 2010–2015. J. Nat. Prod..

[26] Takaki K., Davis J.M., Winglee K., Ramakrishnan L. (2013). Evaluation of the pathogenesis and treatment of Mycobacterium marinum infection in zebrafish. Nat. Protoc..

