# Supplementary material for: Etamycin as a Novel Mycobacterium abscessus Inhibitor
Source: Int J Mol Sci. 2020 Sep 21;21(18):6908. doi: 10.3390/ijms21186908 (PMC7555287; doi:10.3390/ijms21186908)
Supplement: Supplementary file 1 [file ijms-21-06908-s001.pdf]

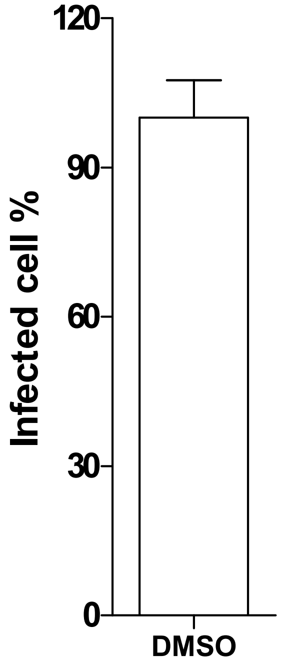

Figure 1. Quantification of mWasabi expressing *M. abscessus* infected cell. mWasabi protein expressing *M. abscessus* subsp. *abscessus* CIP104536^T (S) infected mBMDMs cells was enumerated by CellReporterXpress® Image Acquisition and Analysis Software on day 3 after treatment with DMSO. Data are expressed as the mean ± S.D. of duplicates for each concentration.
